# Virologic characterization of genotype 4 hepatitis C virus variants in patients treated with telaprevir

**DOI:** 10.1186/1743-422X-11-93

**Published:** 2014-05-16

**Authors:** Sandra De Meyer, Anne Ghys, Inge Dierynck, Maria Beumont, Donghan Luo, Gaston Picchio

**Affiliations:** 1 Janssen Infectious Diseases BVBA, Turnhoutseweg 30, B2340 Antwerpen, Beerse, Belgium; 2 Janssen Research and Development, Titusville, NJ, USA

**Keywords:** Genotype 4, HCV, Mutation, Resistance, Telaprevir, Viral kinetics

## Abstract

**Background:**

Study C210 was a Phase IIa, exploratory trial to assess the activity of telaprevir on hepatitis C virus (HCV) early viral kinetics in treatment-naïve patients infected with genotype 4 (G4) HCV.

**Methods:**

Patients were randomized to receive peginterferon and ribavirin alone, telaprevir monotherapy (T arm), or telaprevir in combination with peginterferon/ribavirin (TPR arm) for 15 days, followed by a 46- or 48-week standard treatment phase. The current analysis aimed to characterize the genotype and phenotype of HCV G4 variants emerging during telaprevir treatment.

**Results:**

Five of the 8 (62.5%) patients in the telaprevir (T) arm had viral breakthrough (vBT) during the investigational treatment phase (between baseline and Day 15), compared to no patients in the TPR arm. HCV G4 viral variants with a T54A/T mutation were detected in two of these patients, as well as two other patients with detectable HCV RNA at the end of telaprevir treatment. Emergence of the T54A/T mutation was associated with a 2- to 4-fold decreased susceptibility to telaprevir. All patients with vBT during the investigational treatment phase or with a T54A/T mutation achieved undetectable HCV RNA 12 or 24 weeks after end of treatment with subsequent peginterferon/ribavirin treatment.

**Conclusions:**

In this analysis in G4 HCV-infected patients, more patients in the telaprevir monotherapy arm experienced vBT with resistant variants compared to none with telaprevir combination therapy. The most commonly selected mutation T54A in telaprevir-treated G4 HCV patients was previously described in the context of G1 infection.

**Trial registration:**

The trial was registered with ClinicalTrials.gov (NCT00580801).

## Introduction

Hepatitis C virus (HCV) infection is a significant global health burden, with an estimated global prevalence of approximately 3% [1]. HCV is classified into six different genotypes (G1–6), each with a distinct geographic variation and responsiveness to treatment [2,3] hepatitis C virus genotype 4 (HCV G4) makes up 20% of HCV infections worldwide, but in general is relatively uncommon in the US and Europe [4-6]. However, there is an increase in HCV G4 in certain parts of Europe, probably driven by immigration from Africa and the Middle-East, where HCV G4 is the most common genotype [4].

Telaprevir is a protease inhibitor approved for the treatment of treatment-naïve and treatment-experienced G1 HCV-infected adult patients with compensated liver disease in the US and Europe [7,8]. In telaprevir phase 3 trials, sustained virologic response (SVR) rates were significantly higher with a regimen of 12 weeks of telaprevir in combination with either 24 or 48 weeks of pegylated interferon/ribavirin (Peg-IFN/RBV) than with 48 weeks of Peg-IFN/RBV alone, both in treatment-naïve and treatment-experienced G1 HCV-infected patients [7-9]. During treatment with direct-acting antivirals (DAA) such as telaprevir, there is the potential for selection of preexisting viral variants with decreased susceptibility to DAAs with a similar mechanism of action [10]. In HCV G1 patients a consistent, subtype-dependent resistance profile was observed in patients who did not achieve an SVR with telaprevir-based treatment, with emergence of resistant variants at NS3 amino acid positions V36, T54, R155, and A156 [11,12].

Limited *in vitro* data suggests that telaprevir has activity against HCV G4 [13]. HCV G4 is highly heterogeneous with up to 18 subtypes identified so far [4]. This large variation in subtype can affect disease pathology and also increase the potential for resistance to treatment, making HCV G4 potentially difficult to treat [4,10]. The current standard treatment for HCV G4 infection is 48 weeks of Peg-IFN/RBV, with variable results [4,14,15]. Overall SVR rates have reportedly been higher for patients with HCV G4a compared to HCV G4b [15]. Patients with HCV G4 who do not achieve an SVR with Peg-IFN/RBV currently lack other treatment options.

Study C210 was a Phase IIa trial designed to examine the activity of telaprevir on HCV early viral kinetics in patients with G4 HCV infection. Telaprevir was administered alone or in combination with Peg-IFN/RBV in treatment-naïve G4 HCV-infected patients. In this study, the *in vivo* intrinsic activity of telaprevir monotherapy against HCV G4 was modest; HCV RNA levels decreased slowly between baseline and Day 15 [16]. Larger, more rapid declines were reported for patients who received telaprevir plus Peg-IFN/RBV, suggesting synergy between telaprevir and Peg-IFN/RBV against HCV G4 [16]. Treatment with telaprevir was generally safe and well tolerated [16].

The aim of this sub-analysis of the C210 study was to determine the genotypic and phenotypic characteristics of HCV viral variants emerging in HCV G4 patients who received telaprevir alone or in combination with Peg-IFN/RBV.

## Results

### Patient disposition and baseline characteristics

Patient disposition and baseline characteristics have been reported in detail elsewhere [16]. Briefly, a total of 24 patients (eight per treatment group) were randomized and treated. Baseline demographics and disease characteristics were generally comparable across treatment groups. However, there was some variation between the groups in gender and race; the proportion of males was 50% in peginterferon/ribavirin (PR) arm, 62.5% in the telaprevir (T) arm, and 75% in the telaprevir in combination with peginterferon/ribavirin (TPR) arm, and the proportion of black patients was 25% in the T and TPR arms, and 50% in the PR arm [16]. No enrolled patients had cirrhosis and 54.2% had HCV RNA ≥800,000 IU/mL.

### Antiviral activity in HCV G4-infected patients

A summary of the antiviral activity is shown in Table 1 and has been discussed in detail elsewhere [16]. During the 2-week investigational treatment phase (baseline to Day 15), viral breakthrough (vBT) occurred in 5/8 patients (62.5%) in the T arm and in no patients in the TPR arm. During the standard treatment phase (Week 2 to end of treatment [EOT], in which all patients received Peg-IFN/RBV), vBT occurred in no patients in the T arm and in 2/8 patients (25.0%) in the TPR arm (Table 1).

**Table 1:** Summary of telaprevir antiviral activity in patients infected with G4 HCV

**Antiviral activity**	**Patients infected with G4 HCV**		
	**T (N = 8)**	**TPR (N = 8)**	**PR (N = 8)**
	**n (%)**	**n (%)**	**n (%)**
Virologic response^a^			
By end of TVR/Pbo treatment	0	1 (12.5%)	0
By EOT	7 (87.5)	6 (75.0)	6 (75.0)
Cumulative viral breakthrough			
By end of TVR/Pbo treatment	5 (62.5)	0	0
By EOT	5 (62.5)	2 (25.0)	1 (12.5)
Missing follow-up data^c^	1	0	0
Relapse^b^	1/7 (14.3)	2/6 (33.3)	1/6 (16.7)
SVR24	5 (62.5)	4 (50.0)	5 (62.5)

^a^HCV RNA <25 IU/mL undetectable; ^b^n/N (%) where N = Number of patients with undetectable HCV RNA at EOT; ^c^Patients with undetectable HCV RNA at EOT for which no HCV RNA measurements were available at follow-up Week 24.

*EOT*: End of treatment; *G*: genotype; *HCV*: Hepatitis C virus; *n*: number of patients with observation; *PR*: Peginterferon/ribavirin arm; *T*: Telaprevir monotherapy arm; *T/PR*: Telaprevir plus peginterferon/ribavirin arm; *SVR24*: Sustained virologic response at 24 weeks after last study dose.

Median HCV RNA levels decreased in patients in the T and TPR arm during the investigational treatment phase (from baseline to Day 15) upon commencing telaprevir monotherapy. However, viral levels plateaued or started to increase (vBT) generally after Day 3 in 5/8 patients [16]. Despite this, of the five patients with vBT after 2 weeks of telaprevir monotherapy, four achieved undetectable HCV RNA with subsequent Peg-IFN/RBV treatment (during the standard treatment phase) and three achieved SVR (one patient had missing follow-up Week 24 data but had undetectable HCV RNA at follow-up Week 12).

In addition to the five patients with vBT during telaprevir monotherapy, the three other patients in the T treatment group who did not meet the definition of vBT had detectable HCV RNA at the end of the 15-day investigational phase. Of these patients, all had undetectable HCV RNA at EOT; two then achieved SVR and one relapsed. Three patients in the TPR arm also had detectable HCV RNA at the end of the 15-day investigational phase; all subsequently had undetectable HCV RNA at the end of the standard treatment phase, one achieved SVR and two relapsed.

### Baseline virologic data and early viral kinetics

Baseline sequence data were available for 23 of the 24 randomized patients. The Trugene 5’NC method was used to determine HCV genotype and subtype for study eligibility, with the majority of subjects either infected with HCV genotype 4a (50.0%) or 4c (29.2%) [16]. For virology analyses, samples were also tested for subtype with a more accurate HCV genotyping method based on sequencing of a 329-bp region within NS5B, followed by semi-automatic blast-based alignment to reference sequences. Based on NS5B sequencing the most common HCV G4 genotype was HCV G4a (45.8% of patients), followed by HCV G4d (16.7%) and HCV G4c (12.5%). Subtype distribution was generally similar across the treatment arms (Table 2); 37.5% (3 patients) were infected with HCV G4a at baseline in the T arm and 50.0% (4 patients) had HCV G4a in the TPR arm. Two patients in the T arm had rare genotypes not present in patients in the TPR arm: one patient had HCV G4h and one patient had HCV G4n. Of the five patients who had vBT during the telaprevir/placebo treatment investigational phase, three had HCV G4a, one had HCV G4c and one had HCV G4n. Regarding patients who had detectable HCV RNA viral levels at the end of the telaprevir/placebo treatment investigational phase, two had HCV G4d (who both achieved SVR), and one had HCV G4h (who relapsed).

**Table 2:** Baseline subtype distribution based on NS5B genotyping by treatment arm

**HCV subtype (NS5B)**	**T (N = 8)**	**TPR (N = 8)**	**PR (N = 8)**	**All patients N = 24**
	**n (%)**	**n (%)**	**n (%)**	**n (%)**
4a	3 (37.5)	4 (50.0)	4 (50.0)	11 (45.8)
4c	1 (12.5)	2 (25.0)	0	3 (12.5)
4d	2 (25.0)	2 (25.0)	0	4 (16.7)
4e	0	0	1 (12.5)	1 (4.2)
4f	0	0	1 (12.5)	1 (4.2)
4h	1 (12.5)	0	0	1 (4.2)
4k	0	0	1 (12.5)	1 (4.2)
4n	1 (12.5)	0	0	1 (4.2)
Genotype unknown	0	0	1 (12.5)	1 (4.2)

*N* = Number of patients with data; *n* = Number of patients with that observation.

*HCV*: Hepatitis C virus; *PR*: Peginterferon/ribavirin arm; *T*: Telaprevir monotherapy arm; *T/PR*: Telaprevir plus peginterferon/ribavirin arm.

No patients with available baseline sequences had detectable mutations associated with decreased susceptibility to telaprevir previously described in the HCV G1 genotype. However, a leucine (L) was found at position 36 of the HCV G4 NS3 region in these patients. A valine (V) is generally found at this position in HCV G1 chronically infected patients, while the V36L substitution confers less than 3-fold reduced susceptibility to telaprevir in G1 patients.

**Figure 1:**
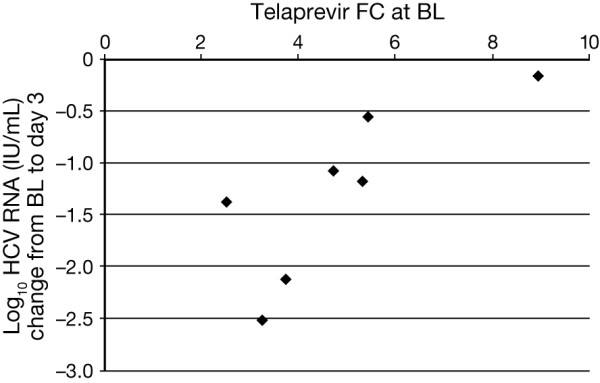
**Scatter plot showing baseline telaprevir susceptibility versus change in HCV RNA from baseline to Day 3 in patients treated with telaprevir monotherapy.** HCV RNA, hepatitis C virus ribonucleic acid; BL, baseline; FC, fold change in IC_50_ value compared to IC_50_ from wild-type (con 1b); IC_50_, 50% inhibiting concentration.

Phenotype data at baseline were obtained for seven patients in the T group and one patient in the TPR group. Telaprevir fold change (FC) at baseline ranged from 2.5 to 8.9 using the replicon-based assay and from 1.7 to 11.9 using the biochemical phenotypic assay. HCV RNA change from baseline to Day 3 versus telaprevir susceptibility at baseline in the seven T patients with data available is shown in Figure 1. For patients in the T arm, there was a correlation between the telaprevir FC at baseline and change in HCV RNA from baseline to Day 3. However these results need to be interpreted with caution due to the low sample size.

### Characterization of viral variants

**Figure 2:**
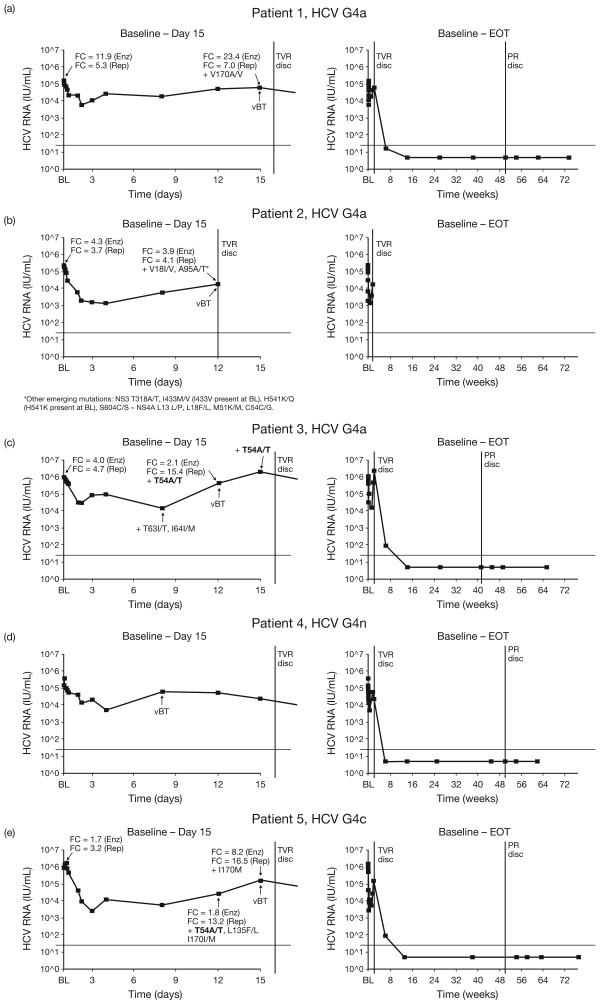
**Viral load profiles of individual patients in the telaprevir monotherapy arm with vBT, for both the investigational and standard treatment phases.** Panels **a**-**e** show data from Patient 1-5. HCV RNA, Hepatitis C virus ribonucleic acid; G, genotype; EOT, end of treatment; TVR disc, telaprevir discontinuation; PR disc, Peginterferon/ribavirin discontinuation; FC, fold change in IC_50_ value compared to IC_50_ from wild-type (con 1b); Rep, replicon-based phenotypic assay; Enz, biochemical –based phenotypic assay. HCV genotype based on NS5B genotyping.

With regards to the five HCV G4-infected patients in the T group who experienced vBT during the investigational phase (baseline to Day 15), Figure 2 shows their individual HCV RNA profiles over time in association with genotypic and phenotypic data. Sequencing was obtained for four out of these five patients. In two patients (patients 3 and 5), emergence of T54A/T, a mutation known to be associated with reduced susceptibility to telaprevir in G1, was detected. In patient 3 (HCV G4a), T54A/T was detected at the point of vBT (Day 12). In patient 5 (HCV G4c), T54A/T was detected at a timepoint before vBT (Day 12), along with two other mutations (L135F/L and I170I/M). In vitro susceptibility to telaprevir was decreased in these two patients with emergence of these variants (Figure 2). In patient 3, FC was 4.0 at baseline and 2.1 on Day 12 using the biochemical phenotypic assay and increased from 4.7 at baseline to 15.4 at Day 12 using the replicon-based assay. In patient 5, FC increased from 1.7 on Day 12 to 8.2 on Day 15 using the biochemical phenotypic assay and from 3.2 on Day 12 to 16.5 on Day 15 using the replicon-based assay. Both patients who had vBT with emerging T54A/T mutations achieved SVR with subsequent Peg-IFN/RBV therapy.

In the other two patients in the T treatment group who had vBT (Patients 1 and 2), and for whom sequencing data were available, no mutations known to reduce telaprevir susceptibility were detected. However, Patient 1 had an emerging V170A/V substitution at the time of vBT (Day 15). An increase in telaprevir FC was noted with the biochemical assay but not the replicon assay (Figure 2a).

A number of other amino acid changes from baseline were identified in patients who experienced vBT in the T or TPR group, including: V18I/V, T63I/T, I64I/M, A95A/T/V and P129A (G4a). These mutations were not consistently seen across patients and no statistical analyses were performed due to the low number of HCV G4 comparator sequences available from non-exposed samples in public or in-house databases.

For the single patient with HCV G4h in the T arm who relapsed after standard therapy, sequence analysis was not available at the time of relapse. However, sequence analysis of the follow-up Week 24 sample did not identify known resistant variants.

Along with Patients 3 and 5 in the T arm who had vBT, emergence of a T54A/T mutation was also detected at Day 15 in a patient in the T treatment group. This patient did not have vBT but the mutation was associated with an 2-fold increase in telaprevir FC compared to baseline using a replicon-based phenotypic assay. One other patient in the TPR group also reported a T54A/T mutation although this was not associated with vBT or any change in telaprevir FC. Both these patients achieved SVR.

Paired phenotype data at baseline and time of vBT/end of telaprevir treatment were available for eight patients in the study. Of these, 3/4 patients with an emerging T54A/T mutation had a 2- to 4-fold increase in telaprevir FC compared with baseline according to the replicon-based phenotypic assay.

## Discussion

Here we report the virologic characterization of variants emerging in the Phase IIa C210 study, the first clinical trial investigating telaprevir in treatment-naïve, chronically-infected HCV G4 patients. Telaprevir combined with Peg-IFN/RBV had greater antiviral activity against HCV G4 than telaprevir monotherapy or Peg-IFN/RBV alone [16]. Telaprevir monotherapy had limited antiviral activity over 15 days; HCV RNA levels decreased only slightly and five of eight patients had vBT (no patients in the TPR group had vBT during this time). The remaining three patients treated with telaprevir monotherapy had detectable HCV RNA at Day 15.

As mentioned above, all vBTs in the investigational, telaprevir treatment phase occurred in the T group. HCV RNA levels only decreased slowly with telaprevir monotherapy compared with combination therapy. These findings are in line with the results of other studies investigating telaprevir monotherapy versus telaprevir plus Peg-IFN/RBV in HCV G1, G2, and G3 patients, and show better results with combination therapy compared to telaprevir monotherapy to control the emergence of resistant variants [10,12,17]. In this regard, 4/5 patients with vBT after 2 weeks of telaprevir monotherapy in our study achieved undetectable HCV RNA with subsequent Peg-IFN/RBV treatment and three achieved SVR (one patient had missing follow-up Week 24 data but had undetectable HCV RNA at follow-up week 12).

Analysis in this study focused on detecting previously reported HCV G1 amino acid mutations in the NS3•4A region; including single change V36A/M, T54A, R155I/K/M/T, and A156S which have been associated with lower level in vitro resistance to telaprevir (3- to 25-fold increase in replicon 50% IC_50_), and single change A156T/V and double change at positions V36M + R155K which have been associated with higher level *in vitro* telaprevir resistance (>25-fold increase in replicon IC_50_) [10,18,19]. None of the 23 patients with sequences obtained by population sequencing in this study had mutations associated with decreased telaprevir susceptibility at baseline, although a leucine (L) substitution in HCV G4 patients was detected in place of a V present in HCV G1 patients at position 36. The functional ramifications of this substitution are as yet unknown, though the V36L substitution in HCV G1 has been shown to exhibit a 2-fold change in telaprevir susceptibility [12].

Sequences were available for four of the five patients who had vBT during the investigational phase of the study (baseline to Day 15). In two of these patients, T54A/T emerged, which is a mutation known to moderately (FC ~6) increase telaprevir resistance in patients with G1 HCV [10]. Two other T54A/T mutations were also detected, one in a patient in the T group who did not have vBT but who had detectable levels of HCV RNA at the end of telaprevir treatment, and one in a patient in the TPR group who also had detectable levels of HCV RNA at the end of telaprevir treatment. Overall, paired phenotype data at baseline and time of vBT or end of telaprevir treatment indicated that the emergence of the T54A/T mutation in this study population was associated with a 2- to 4-fold increase in telaprevir FC compared with baseline. Importantly, this mutation was not associated with failure of subsequent Peg-IFN/RBV treatment and all patients with a T54A/T mutation subsequently achieved SVR.

Taken together, these findings suggest that vBT and the emergence of resistant variants following telaprevir monotherapy had little or no impact on the efficacy of subsequent treatment with Peg-IFN/RBV. However, additional data on the clinical relevance of resistance with DAAs are required. In this regard, interim results from EXTEND, a multinational, 3-year follow-up study of patients treated with telaprevir-based regimens in Phase II and Phase III clinical trials, showed that, after ceasing telaprevir treatment, levels of resistant variants can rapidly decline as they are outcompeted by other, potentially more fit wild-type variants [20]. Additional research is needed to ascertain whether these patients can be successfully retreated with the same DAA class. Nevertheless, the presence of some mutations can still be detected years later [20], and it is therefore important to limit potential for resistant variants [11]. The rationale for combination therapy is due to the non-competing mechanisms of actions of the different agents used, which means that viruses associated with resistance to one class of agent are often susceptible to another class [11]. This is supported by data in this study, as no variants known to be associated with resistance to telaprevir were detected in patients treated with TPR therapy.

Several other mutations were also detected in HCV G4 infected patients in this study. The variant V170A (HCV G4a) was observed in one patient at the same time as vBT in the T arm. This mutation has been previously observed in the HCV G1 population, although not at high frequency. It has also been linked with low-to-moderate levels of resistance to boceprevir [10,21]. Another patient (HCV G4c) had three mutations detected at the time of vBT (including I170I/M and T54A/T). However, the importance of the combination of these mutations is not well defined and needs to be investigated further. Other mutations identified as emerging from baseline in patients with vBT were: V18I, T63I, I64M, A95T/V, P129A, and L135F. Due to the limited sample size, the lack of consistency of the mutations across patients and the scarcity of HCV G4 comparator sequences, the relevance of these mutations is currently not understood. Consequently, on the basis of this study, new HCV G4-specific mutations could not be identified.

Although few mutations known to be associated with reduced susceptibility to telaprevir in HCV G1 were observed in this study, this may be due to sample size limitations. Other viral mutations that affect DAA activity in HCV G1 may also be relevant for patients with HCV G4, since active site residues in HCV G1 and HCV G4 in NS3 protease domains are similar. Genotypic polymorphisms in the HCV NS3•4A proteases inter- and intra-genotypes are in general located on the protease surface, far from the telaprevir binding site [22].

Telaprevir FC at baseline ranged from 2.5 to 8.9 using the replicon-based assay and from 1.7 to 11.9 using the biochemical phenotypic assay. A correlation was observed between the telaprevir FC at baseline and change in HCV RNA from baseline to Day 3. However, as mentioned below, this finding needs to be interpreted with caution.

Conclusions inferred from the result of this study are limited due to small sample size and insufficient power to perform appropriate statistical analysis. The small population of this study also means that discrepancies in baseline data (i.e., gender and race) may exert an effect on study results, along with baseline differences in HCV G4 subtype between treatment groups. Unfortunately, sequencing and virologic response data could not be obtained for some patients in this study, further compounding the issue of small sample size. Despite these limitations, it should be noted that the size of this study is not unusual for an exploratory, proof-of-principle study investigating molecular consequences of antiviral activity.

In summary, the results of this study indicates that the *in vivo* intrinsic activity of telaprevir monotherapy against genotype 4 HCV is modest after 2 weeks of treatment, and suggests synergy between telaprevir and Peg-IFN/RBV against genotype 4 HCV compared with Peg-IFN/RBV alone. Whether the synergy between telaprevir and Peg-IFN/RBV translates in higher SVR rates than Peg-IFN/RBV alone or not, needs to be further investigated. During the telaprevir/placebo treatment phase of this study, all cases of vBT occurred with telaprevir monotherapy, highlighting the importance of combination TPR therapy. Despite this, most patients who experienced vBT with telaprevir monotherapy achieved SVR, indicating that vBT and the emergence of resistant variants do not preclude successful Peg-IFN/RBV treatment. The most common mutation selected in G4 patients during telaprevir therapy was T54A, a mutation previously described for telaprevir in the context of G1 infection.

## Materials and methods

The methodology for the C210 exploratory study, including full exclusion and inclusion criteria, have been described elsewhere [16]. This study was a Phase IIa, partially blinded, randomized, multiple-dose trial conducted from January 2008 until January 2010 at a single French center in male or female, treatment-naïve patients, aged 18–65 years, who were chronically infected with HCV G4. The study was approved by the center’s institutional review board and conducted in accordance with the Declaration of Helsinki and Good Clinical Practice guidelines (CPP Ile De France VI, 10 Pavillon Jacquart, 47 Boulevard de l’hopital, 75013 Paris, France). Written informed consent was obtained from all patients. The trial was registered with ClinicalTrials.gov (NCT00580801).

### Interventions

The trial consisted of a screening period of a maximum of 6 weeks, a 2-week investigational phase, a 46- or 48-week standard treatment phase, and a follow-up period of 24–48 weeks. Patients were randomized in a 1:1:1 ratio to one of three treatment arms: during the investigational phase, patients received telaprevir 750 mg every 8 hours (q8h) alone (T arm), telaprevir plus Peg-IFN alfa-2a (180 μg once weekly) and weight-based RBV 1,000–1,200 mg/day (TPR arm), or placebo q8h plus Peg-IFN/RBV (PR arm). During the standard treatment phase, all patients received Peg-IFN/RBV for 48 weeks (T arm) or 46 weeks (TPR and PR arms). The present analysis focused on the T and TPR arms since the primary goal was to characterize HCV variants detected in telaprevir-treated patients infected with G4 HCV.

Primary and secondary endpoints of this study have been reported elsewhere [16]. Patients were considered to have viral breakthrough vBT if they had a confirmed increase >1 log_10_ in HCV RNA concentration from the lowest level reached or a confirmed value of HCV RNA >100 IU/mL in patients whose HCV RNA had previously reached <25 IU/mL. Patients were considered to have had a relapse when they had confirmed detectable HCV RNA during the follow-up period, despite having undetectable HCV RNA at EOT. vBT and relapse were considered confirmed when respective criteria were fulfilled at two or more consecutive time points or at the last observed time point in case of trial termination. For patients in the T arm, no confirmation of vBT was needed during the investigational treatment phase. SVR was defined as having undetectable HCV RNA at EOT through to 24 weeks after the last dose of study medication.

### Virologic assessments

Samples for viral sequencing of the NS3•4A region were obtained at baseline, Days 2, 3, 4, 8, 12, and 15, Weeks 4, 12, 24, 36, and EOT. If HCV RNA was undetectable at EOT, then further samples were taken at Weeks 4, 8, 12, and 24. Otherwise, samples were taken at Weeks 4 and 24 after EOT. Samples were also obtained 24 weeks after relapse was detected.

HCV RNA levels were evaluated using COBAS® Taqman® HCV test version 2.0, with a limit of quantification of 25 IU/mL. If an HCV RNA signal was detected at <25 IU/mL it was reported as “<25 IU/mL detected”, if no HCV RNA was detected in the sample it was reported as “<25 IU/mL, target not detected”. HCV RNA “<25 IU/mL, target not detected” is also described as undetectable HCV RNA in the manuscript.

### Genotypic analyses

For virology analyses, HCV genotype and subtype were determined based on sequencing information from the NS5B gene (Table 2). Subtype-specific population-based HCV NS3 amplification and sequencing protocols (Virco BVBA, Beerse, Belgium) were performed on patient plasma samples to determine the predominant HCV genotype in all baseline samples, and also in post-baseline samples from telaprevir-treated patients who did not achieve an SVR or who had vBT. The lower limit of detection of the sequencing assay was approximately 1,000 IU/mL HCV RNA.

Population-based analysis focused on detecting previously reported HCV G1 amino acid mutations in the HCV G4 subtype specific NS3 region (amino acids 1–181). This included detection of single change V36A/M, T54A, R155I/K/M/T, A156S/T/V and double change at positions V36M + R155K [12,19].

### Phenotypic analyses

Two types of assay were used to conduct phenotypic analyses: a biochemical assay and a transient replicon-based assay.

Regarding the biochemical assay, selected patient-derived NS3 (amino acid 1–181) sequences were cloned into a modified pET11a vector and expressed using *Escherichia coli* Rosetta2 (DE3) (Novagen, Madison, WI, USA). *In vitro* telaprevir inhibition of NS3•4A was determined using a fluorescence-resonance energy transfer cleavage assay with retS1 peptide substrate (Anaspec, San Jose, CA, USA). Inhibition values derived from a series of telaprevir concentrations were used to calculate the 50% inhibitory concentration (IC_50_) value. Fold change in IC_50_ values (FC) compared with HCV wild-type (Con1b) were also calculated.

For the transient replicon-based assay, derived protease sequences were shuttled into a G1b shuttle replicon with the corresponding protease sequence deleted. Pooled colonies containing several chimeric replicons were then collected and DNA was *in vitro* transcribed using a T7 MEGAscript High Yield Transcription Kit (Ambion, Foster City, CA, USA). Purified wild-type or replicated RNA was then transfected into human hepatoma Huh-7 lunet cells, and incubated in 384-well plates containing telaprevir dilution series. After incubation, luminescence based dose–response curves were used to determine IC_50_ and FC was calculated as IC_50_ of shuttle construct divided by IC_50_ of wild-type G1b replicon (mean value of three experiments).

### Statistical analyses

Because of the low number of patients infected with HCV G4 in this study, no statistical analysis was performed and data were summarized descriptively.

## Abbreviations

BL: Baseline; DAA: Direct-acting antiviral; EOT: End of treatment; FC: Fold change in IC50; HCV: Hepatitis C virus; HCV G4: Hepatitis C virus genotype 4; IC50: 50% Inhibitory concentration; L: Leucine; Pbo: Placebo; Peg-IFN: Peginterferon; PR: Peginterferon/ribavirin arm; q8h: Every 8 hours; RBV: Ribavirin; SVR: Sustained virologic response; T: Telaprevir monotherapy arm; TPR: Triple combination therapy arm; TVR: Telaprevir; V: Valine; vBT: Viral breakthrough.

## Competing interests

All authors are employees of Janssen Pharmaceuticals and may be Johnson & Johnson stockholders. The authors declare that they have no competing interests.

## Authors’ contributions

All authors have been involved in drafting the manuscript and/or revising it critically for important intellectual content. All authors have provided final approval of the manuscript for publication.
